# High polygenic risk score for exceptional longevity is associated with a healthy metabolic profile

**DOI:** 10.1007/s11357-022-00643-y

**Published:** 2022-08-16

**Authors:** Mary Revelas, Anbupalam Thalamuthu, Anna Zettergren, Christopher Oldmeadow, Jenna Najar, Nazib M. Seidu, Nicola J. Armstrong, Carlos Riveros, John B. Kwok, Peter R. Schofield, Julian N. Trollor, Margda Waern, Margaret J. Wright, Henrik Zetterberg, David Ames, Kaj Belnnow, Henry Brodaty, Rodney J. Scott, Ingmar Skoog, John R. Attia, Perminder S. Sachdev, Karen A. Mather

**Affiliations:** 1 Centre for Healthy Brain Ageing, School of Psychiatry, UNSW Medicine & Health, UNSW, Sydney, Australia; 2 Neuroscience Research Australia, Sydney, NSW Australia; 3 Neuropsychiatric Epidemiology Unit, Department of Psychiatry and Neurochemistry, Institute of Neuroscience and Physiology, the Sahlgrenska Academy, Centre for Ageing and Health (AGECAP) at the University of Gothenburg, Gothenburg, Sweden; 4 Hunter Medical Research Institute, Newcastle, NSW Australia; 5 Region Västra Götaland, Sahlgrenska University Hospital, Psychiatry, Cognition and Old Age Psychiatry Clinic, Gothenburg, Sweden; 6 Mathematics and Statistics, Curtin University, Perth, Australia; 7 Queensland Brain Institute, University of Queensland, Brisbane, Australia; 8 School of Medical Sciences, UNSW, Sydney, Australia; 9 Department of Developmental Disability Neuropsychiatry, School of Psychiatry, UNSW Medicine & Health, UNSW, Sydney, Australia; 10 Region Västra Götaland, Sahlgrenska University Hospital, Psychosis Clinic, Gothenburg, Sweden; 11 Centre for Advanced Imaging, University of Queensland, Brisbane, Australia; 12 Department of Neurodegenerative Disease, UCL Institute of Neurology, London, UK; 13 UK Dementia Research Institute at UCL, London, UK; 14 Department of Psychiatry and Neurochemistry, Institute of Neuroscience and Physiology, the Sahlgrenska Academy at the University of Gothenburg, Mölndal, Sweden; 15 Clinical Neurochemistry Laboratory, Sahlgrenska University Hospital, Mölndal, Sweden; 16 Hong Kong Centre for Neurodegenerative Diseases, Hong Kong, China; 17 University of Melbourne Academic Unit for Psychiatry of Old Age, St George’s Hospital, Kew, VIC Australia; 18 National Ageing Research Institute, Parkville Victoria, Australia; 19 Dementia Centre for Research Collaboration, UNSW, Sydney, Australia; 20 Faculty of Health and Medicine, University of Newcastle, Callaghan, NSW Australia; 21 Pathology North, John Hunter Hospital, Newcastle, NSW Australia; 22 Neuropsychiatric Institute, Prince of Wales Hospital, Randwick, NSW Australia

**Keywords:** Longevity, PRS, Metabolic syndrome

## Abstract

Healthy metabolic measures in humans are associated with longevity. Dysregulation leads to metabolic syndrome (MetS) and negative health outcomes. Recent exceptional longevity (EL) genome wide association studies have facilitated estimation of an individual’s polygenic risk score (PRS) for EL. We tested the hypothesis that individuals with high ELPRS have a low prevalence of MetS. Participants were from five cohorts of middle-aged to older adults. The primary analyses were performed in the UK Biobank (UKBB) (*n* = 407,800, 40–69 years). Replication analyses were undertaken using three Australian studies: Hunter Community Study (*n* = 2122, 55–85 years), Older Australian Twins Study (*n* = 539, 65–90 years) and Sydney Memory and Ageing Study (*n* = 925, 70–90 years), as well as the Swedish Gothenburg H70 Birth Cohort Studies (*n* = 2273, 70–93 years). MetS was defined using established criteria. Regressions and meta-analyses were performed with the ELPRS and MetS and its components. Generally, MetS prevalence (22–30%) was higher in the older cohorts. In the UKBB, high EL polygenic risk was associated with lower MetS prevalence (OR = 0.94, *p* = 1.84 × 10^–42^) and its components (*p* < 2.30 × 10^–**8**^). Meta-analyses of the replication cohorts showed nominal associations with MetS (*p* = 0.028) and 3 MetS components (*p* < 0.05). This work suggests individuals with a high polygenic risk for EL have a healthy metabolic profile promoting longevity.

**Supplementary Information:**

The online version contains supplementary material available at 10.1007/s11357-022-00643-y.

## Introduction

Owing to factors such as improved healthcare and favourable lifestyle choices, human lifespan has increased significantly over the last century with many individuals in developed countries surpassing 80 years of age [1]. As the proportion of older adults escalates, it necessitates an increased understanding of the factors promoting healthy ageing. The World Health Organization has defined healthy ageing “as the process of developing and maintaining the functional ability that enables wellbeing in older age” [2]. Cognisance of the factors involved in successful ageing is crucial to developing strategies to promote high quality of life in older adults and lessening the medical, social and economic burden associated with caring for older adults with ageing-related decline and disease.

Exceptional longevity (EL) is often associated with healthy ageing as many centenarians markedly compress the onset and duration of illnesses to the very end of their life [3]. EL is multifactorial, with genetics playing some role; family and twin studies estimate heritability of lifespan as low to moderate (20–35%) [4], with the genetic contribution increasing later in life (> 60 years) [5]. A number of studies have identified genetic factors associated with longevity [6, 7]. A review and meta-analysis of the genetic variants suggested that genes related to cardiovascular health may be implicated in exceptional longevity [8]. Studies of extreme longevity have shown that siblings of centenarians have between an 8 (female) to 17 (male) times higher chance of reaching 100 years of age when compared to birth year matched population controls [9]. More recent work on a unique dataset comprising individuals (*n* = 2070) that had survived to the oldest 1% for their birth year cohort revealed new rare variants in chromosomes 4 and 7 that associated with extreme longevity [10]. Additional analysis on 28,297 participants from several independent longevity studies showed *APOE* ε2 and ε4 allele specific effects on exceptional longevity [11]. Furthermore, studies in long-lived families identified a heritable (*h*^2^ = 0.40, *p* < 0.001) healthy metabolic profile, comprised of age and gender adjusted z-scores for favourable fasting levels of glucose, insulin, triglycerides, high-density lipoprotein cholesterol, body mass index, waist circumference, interleukin-6 and high-sensitivity C-reactive protein [12]. Additional evidence of the importance of metabolic health in longevity was demonstrated by a recent study that identified two candidate longevity genes, *FN3KRP* (protein glycation) and *PGP* (glycerol-3 phosphate regulation), which are both involved in metabolic pathways [13].

Healthy metabolic measures in humans are associated with longevity. Dysregulation of these measures may lead to development of the metabolic syndrome (MetS) which has been defined as the presence of cardiovascular risk factors (e.g. dyslipidaemia, obesity and hypertension [14]). The relationship of MetS with longevity is of particular interest due to its increased prevalence with ageing [15]; raising the question as to whether exceptionally, long-lived individuals have a different incidence of MetS.

In support of this hypothesis, a recent meta-analysis examining longevity genetic polymorphisms found that all significant variants were located in genes that can be linked to cardiovascular phenotypes and the development of MetS (*APOE, FOXO3A*, *ACE, Klotho* and *IL6*) [8]. One of the key components of MetS is insulin resistance and the genetic variants identified in the longevity meta-analysis for *Klotho*, *IL6* and *FOXO3A* inhibit the insulin/IGF-1 signalling pathway [16–18]. Decreased insulin sensitivity has been recognised as a hallmark of ageing and is evolutionarily conserved from model organisms to humans [19]. Studies of centenarians and their offspring have reported their ability to preserve glucose homeostasis and insulin sensitivity despite ageing [20, 21]. Another study of offspring from nonagenarian siblings (*n* = 121) observed both a lower prevalence of MetS as well as an improved glucose tolerance when compared to their partners (*n* = 113) [22].

Healthier adipocytokine (cytokines secreted by adipose tissue) profiles have been reported in centenarians [23]. Dysregulation of adipocytokine appears to play a central role in adiposity, MetS, obesity and cardiovascular disease. A study of 118 long-lived individuals (aged ≥ 95 years) and their offspring (*n* = 228) observed increased levels of an adipocytokine – adiponectin – a key metabolic regulator [24] compared to younger unrelated controls (*n* = 78). Adiponectin was (i) inversely correlated with body mass index (BMI) and waist circumference and (ii) positively correlated with high-density lipoprotein (HDL) and lipoprotein particle size in this study [25]. Additionally, functional polymorphisms in the obesity-related leptin and leptin receptor genes have been previously associated with longevity in centenarians (*n* = 128) compared to younger controls (*n* = 414) [26].

Another component of MetS—dyslipidaemia, is characterised by increased triglyceride (TG) and/or low-density lipoprotein (LDL) levels and decreased HDL levels. Notably, the genetic variant with the largest effect size in the above-mentioned longevity genetic meta-analysis is the ε4 allele of the apolipoprotein E (*APOE)* gene [8]. The product of this gene plays a critical role in lipoprotein metabolism, with imbalances of the protein linked to Alzheimer’s disease, obesity, diabetes, cardiovascular disease and an increased risk of MetS [27, 28]. A study evaluating plasma apolipoproteins in 1067 individuals (aged 56–105) observed that centenarians had the highest plasma ApoE levels and the lowest frequency of *APOE* ε4 allele carriers in comparison to younger groups [29]. Furthermore, a unique lipoprotein profile has been suggested to promote healthy ageing and exceptional longevity [30]. A healthier lipid profile, larger LDL particle size and low triglyceride levels, in the offspring of nonagenarians compared to the offspring’s partners was observed in the Leiden Longevity Study [31].

Further studies examining genetic vascular factors involved in human longevity have suggested associations with genes involved in blood pressure regulation (*PON1* and *PAI-1)* [32]. A recent investigation observed a lower incidence of hypertension in Portuguese centenarians (*n* = 253) who also had a higher frequency of pro-longevity variants in two vascular-related genes, *ACE* and *NOS3,* compared to controls, underscoring the importance of healthier cardiovascular risk profiles in achieving longevity [33]. In fact, MetS has demonstrated to promote arterial ageing as well as exaggerate hypertensive-related changes in both men and women [34, 35].

Genetic studies on exceptional longevity have provided evidence that metabolism-related pathways play important roles in attaining long life, such as the EL meta-analysis described above [8]. Several genome-wide association studies (GWAS) on EL have been performed [36, 37]. Deelen et al. found EL was genetically correlated with coronary artery disease (CAD), type 2 diabetes and paternal age at death [38]. Furthermore, they found that three components of MetS (HDL-cholesterol, triglycerides & waist circumference) were also significantly genetically correlated to longevity (both the 90^th^ and the 99^th^ percentile of survival meta-analyses) after adjustment for multiple testing. This once again illustrates the importance of metabolism-related pathways in EL. Moreover, the availability of prior EL GWAS summary statistics allows the calculation of an individual’s polygenic risk score (PRS) for EL, which can be used for further studies.

The aim of this study was to assess if a high PRS for EL is associated with a low prevalence of MetS and its components. This study was undertaken in five population-based independent and varied cohorts, ranging in age from 40 to 93 years with genetic and metabolic data available, with an overall large sample size of over 400,000 participants of European ancestry.

## Materials and Methods

**Fig. 1:**
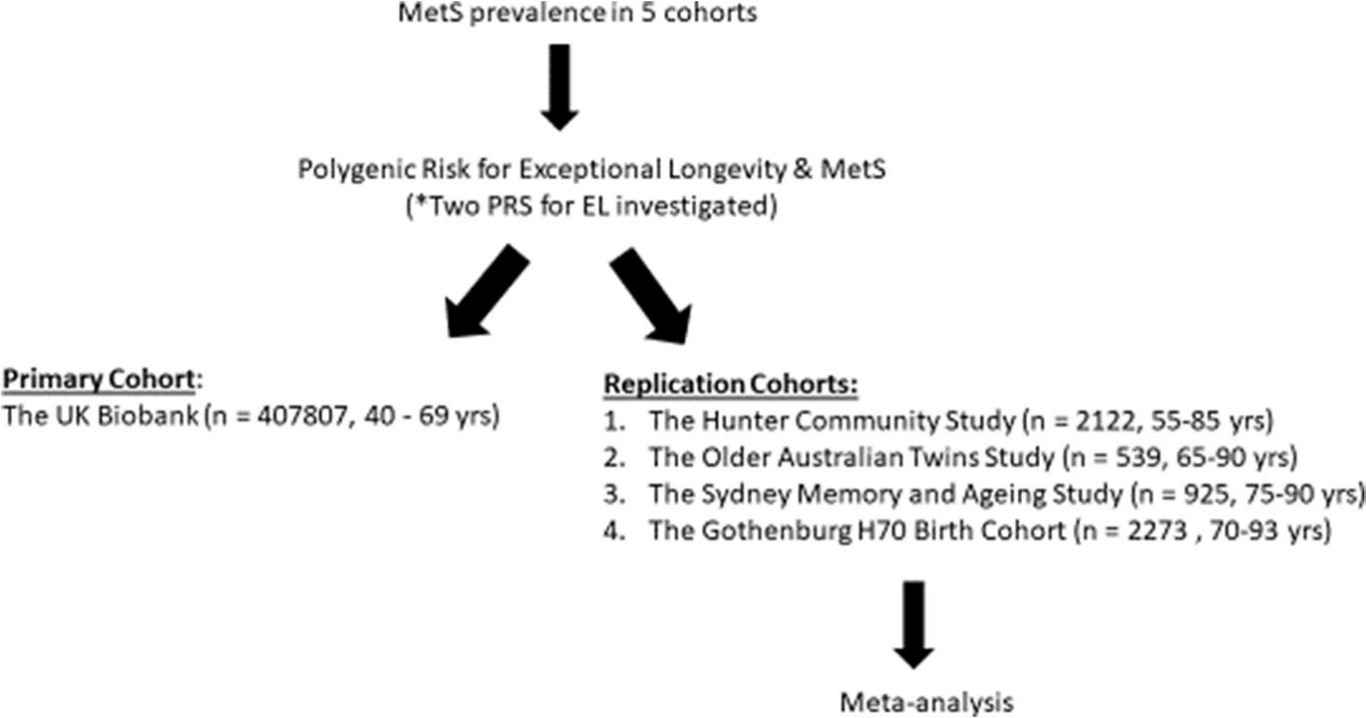
Flow diagram of the analysis plan for the investigation of polygenic risk for exceptional longevity with metabolic syndrome

Please refer to Fig. 1 for a flow diagram that outlines the research plan.

### Participants

For each study, participants with both genetic and phenotypic data were utilised, thus reducing the initial sample size.

#### Primary cohort: UK Biobank (UKBB)

This study is comprised of 502 631 individuals (age range 40–69, mean age 56.5 years, 46% male, Application Number 53850) enrolled at 22 assessment sites in England, Scotland and Wales between 2006 and 2010 [39]. Individuals were recruited via the National Health Register (NHS) and written informed consent was obtained. Extensive phenotypic data were collected at baseline including information from questionnaires, physical measurements, sample assays, imaging as well as mortality records. The North West Multi-centre Research Ethics Committee approved the UKBB study. Blood samples were collected for DNA and blood biochemistry analysis.

#### Replication cohorts

##### Hunter Community Study (HCS)

HCS is a cohort of 3253 individuals (age range 55–85, mean age 66.3 years, 46% male) recruited from the Hunter region of NSW using the compulsory electoral roll and Medicare lists from New South Wales (NSW), Australia [40]. The University of Newcastle and Hunter New England Human Research Ethics Committees (HREC 03/12/10/3.26) approved the HCS.

##### Older Australian Twins Study (OATS)

OATS recruited mono and dizygotic twins 65 years and older via the Australian Twin Registry (ATR), a national registry of twins willing to participate in health research. OATS is a cohort of 623 individuals (age range 65–90, mean age 70.7 years, 40% male) [41]. Ethics approval was obtained from the following ethics committees: Australian Twin Registry, University of New South Wales, University of Melbourne, Queensland Institute of Medical Research and the South Eastern Sydney & Illawarra Area Health Service.

##### Sydney Memory and Ageing Study (Sydney MAS)

Sydney MAS recruited participants using the compulsory electoral roll and Medicare lists from New South Wales (NSW), Australia [42]. Sydney MAS is a cohort of 1037 individuals (age range 70–90 years, mean age 78.8 years, 45% male). The Human Research Ethics Committees of UNSW Sydney and the South Eastern Sydney and Illawarra Area Health Service (HC05037, HC09382, HC14327 and HC190962) granted ethics approval for Sydney MAS.

Written informed consent was obtained from all participants. Fasting blood samples were collected for DNA and blood biochemistry analysis. More information on the Australian studies are found in McEvoy et al. and Sachdev et al. [40–42].

##### The Gothenburg H70 Birth Cohort Studies

These studies recruit participants based on specific birth dates obtained from the Swedish Tax Agency’s population register and registered as residents in Gothenburg [43]. Included in this specific study sample were 2273 individuals (age range 70–93 years, mean age 73.3, 38% male) who were born in 1908, 1914, 1922 and 1930 and had an examination in 2000–2002, 2005–2007, 2009–2011 or 2015–2017, and individuals who were born 1944 and examined in 2014–2016, with both genetic data and information on all metabolic variables. Consent forms were obtained before undergoing extensive examinations at the Neuropsychiatric outpatient department at the Sahlgrenska University Hospital, or at the residence of the participant for those who had difficulties to come to the clinic, including interviews and physical examinations. The interviews included questions on present and past medical history and social factors with the participant and with a close informant. The physical examinations included blood sampling, anthropometric measures (e.g., height and weight) and blood pressure. The Regional Ethical Review Board in Gothenburg approved the Gothenburg H70 Birth Cohort Studies.

### MetS calculation

Blood biochemical measures used for calculation of MetS were serum glucose, lipids—cholesterol, triglycerides, high-density lipoproteins. For the UKBB, a Beckman Coulter AU5800 measured serum glucose, HDL-cholesterol and triglycerides, by standard methods. Since blood samples were collected at different times of fasting in the UKBB, glucose and triglyceride levels were modified as per Lind [44]. Specifically, glucose levels were adjusted down by 1.5 mmol/L if reported fasting time was 0 h, 3.0 mmol/L if fasting was 1 h, 1.0 mmol/L if fasting was 2 h, 0.3 mmol/L if fasting was 3 h and no correction if fasting time was > 3 h. For triglycerides, the levels were adjusted down by 0.1 mmol/L if reported fasting time was 1 h. Similarly, reductions were 0.2, 0.4, 0.6, 0.65, 0.4 and 0.1 mmol/L for fasting times 2–7 h. [44]. The H70 study cohort and the Australian studies collected fasting blood samples and hence measurements were not adjusted for glucose and lipids. Blood pressure was measured twice in the sitting position with the automated Omron device and the average was used. For the H70 study cohort, blood pressure was measured in the same manner but only once. In all cohorts, a trained researcher or a research nurse measured waist circumference. Medication was self-reported.

The harmonized National Cholesterol Education Program (NCEP) criteria for MetS based on Lind were used [44]. A participant had MetS if three or more of the following five criteria (based on 8 components) were met: systolic blood pressure ≥ 130 and/or diastolic blood pressure ≥ 85 mmHg and/or currently using antihypertensive medication/s, serum glucose ≥ 6.1 mmol/L or using antidiabetic treatment, serum triglycerides ≥ 1.7 mmol/L, HDL-cholesterol < 1.0 mmol/L in men and < 1.3 mmol/L in women and waist circumference > 102 cm in men and > 88 cm in women. If three or more of the five criteria had missing values for a given participant, MetS status was set as missing.

### Genotyping

In the UKBB cohort, DNA was extracted using standard protocols. Genotyping was performed using the Affymetrix UK BiLEVE Axiom® array on an initial 50 000 participants according to the manufacturer’s instructions. The remaining 450 000 participants were genotyped using the Affymetrix UK Biobank Axiom® array, that genotyped ~ 850 000 variants. The two arrays have over 95% common content. Genotyped SNPs were removed if as follows: (i) quality control failed in more than one batch (ii) overall missing rate was ≥ 5% and (iii) minor allele frequency was < 0.01. Samples identified as outliers for heterozygosity and missing rate were excluded. The quality controlled genotype data was phased and imputation was carried out with the IMPUTE4 program (https://jmarchini.org/software/) [45] using the Haplotype Reference Consortium reference panel [46] and UK10K haplotype resources [47] by a collaborative group headed by the Wellcome Trust Centre for Human Genetics. The result of the imputation process is a dataset with ~ 96 million SNPs in 487 442 individuals.

The data filtering steps utilised for the current analysis are as follows: (i) selected Caucasians (*n* = 409 615), (ii) removal of participants with poor heterozygosity or missing data (*n* = 480), (iii) exclusion of participant self-declared as having a mixed ancestral background (*n* = 692) and high heterozygosity rate (after correcting for ancestry) or high missing rate (*n* = 840), (iv) participant excluded from kinship inference process, meaning having ten or more third degree relatives identified (*n* = 188) and (v) lastly, removal of outliers for heterozygosity or missing rate (*n* = 968). The final filtered sample size for this analysis is 407 807 from the total sample size of 502 505 participants.

In all of the Australian cohorts, DNA was extracted using standard methods. HCS were genotyped using the Affymetrix Axiom Kaiser array, OATS using the Illumina Omni Express array and Sydney MAS samples using the Affymetrix Genome-wide Human SNP Array 6.0 all according to the manufacturers’ instructions. Genotyped SNPs were excluded if the following criteria were observed: (i) the call rate was < 95%, (ii) *p*-value for HWE was < 10^–6^ and (iii) minor allele frequency was < 0.01 (iv) strand ambiguous (A/T and C/G). If first or second-degree relatives were identified, only one family member was retained for analysis. EIGENSTRAT analysis allowed for the detection and removal of any ethnic outliers. Any samples with deviations from heterozygosity were also omitted (FHET outside + / − 0.2).

After QC checks, there was data on 739,276 SNPs for HCS. OATS and Sydney MAS had genotyping data on 636,749 and 734,550 SNPs respectively. The quality controlled genotype data was imputed in the Michigan imputation server (https://imputationserver.sph.umich.edu) [48] using the Haplotype Reference Consortium reference panel (v3.20101123), and SNPs with high quality (imputation quality score > 0.6) were used in the calculation of the ELPRS. The genotypes for the *APOE* single nucleotide polymorphisms (SNPs) rs7412 and rs429358 were extracted from the imputed dosage using PLINK. Both SNPs were imputed with high accuracy in both of the cohorts (*R*^2^ > 0.80) and *APOE* ε2/3/4 haplotypes were inferred [49]. The final filtered sample size for this analysis is 2122, 539 and 925 participants for HCS, OATS and MAS respectively.

In the Gothenburg H70 Birth Cohort Studies, extraction of DNA from whole blood was performed according to standard procedures at LGC Genomics in Berlin (Germany). All the DNA samples have been genotyped at University College London (UK), using the Neuro Consortium Array (neurochip) from Illumina [43]. QC included the removal of individuals due to any of the following: per-individual call rate < 98%, sex mismatch and deviation from heterozygosity (FHET outside + / − 0.2). Furthermore, individuals were defined as non-European ancestral outliers, and removed, if their first two PCs exceeded 6 standard deviations from the mean values of the European samples in the 1000 Genome global reference population. Closely related individuals were removed based on pairwise PI_HAT (i.e. proportion of the genome that is in identity-by-descent; calculated using genome option in PLINK) >  = 0.2. Genetic variants were excluded due to per-SNP call rate < 98%, minor allele frequency (MAF) < 0.01, and Hardy–Weinberg disequilibrium (*p* < 1 × 10^–6^). The Sanger imputation service was used to impute post-QC, using the reference panel of Haplotype Reference Consortium data (HRC1.1) [50]. The single nucleotide polymorphisms (SNPs) rs7412 and rs429358, defining the *APOE* alleles *ε2*, *ε3* and *ε4*, were also genotyped, using the KASPar PCR SNP genotyping system (LGC Genomics, Hoddesdon, Herts, UK). The final filtered sample size available for this specific study (i.e. individuals with both genetic and metabolic data) was 2273 participants.

### Exceptional longevity polygenic risk score (ELPRS)

ELPRS were generated by using a Python package for polygenic risk score with continuous shrinkage (PRS-CS) available on the github repository (https://github.com/getian107/PRScs). PRS-CS infers posterior SNP effect sizes under continuous shrinkage priors using summary statistics obtained from a GWAS. In contrast to using GWAS *p*-value thresholds, PRS-CS uses the results from all SNPs in the summary statistics rather than a subset. This allows for considerable computational improvements owing to marker-specific adaptive shrinkage, which is robust to varying genetic architectures, and it enables multivariate modelling of local LD patterns [51]. PRS-CS was run with default parameters (gamma-gamma priors PARAM_A = 1, PARAM_B = 0.5, shrinkage parameter phi = 0.2, MCMC iterations = 1000, number of MCMC burn-in iterations = 500 and the MCMC thinning factor = 5). For the primary analyses of this study, the summary statistics were from an EL GWAS study, based on cases who had lived to an age above the 90^th^ percentile for survival and controls were those who had died at or before the 60^th^ percentile [38]. Due to the strong associations between exceptional longevity and the *APOE* locus [8], the ELPRS were also calculated after removing this locus (chromosome 19, base pair 45,116,911 – 46,318,605, Genome Reference Consortium Human Reference Build 37).

Secondary analyses also examined additional PRSs using the UKBB and the Australian cohorts. Firstly, a PRS was calculated using the summary statistics from the same EL GWAS as above but using cases who had lived to an age above the 99^th^ percentile for survival. Secondly, a PRS was calculated based on 330 variants associated with longevity [52], based on data from the PGS Catalog (http://www.pgscatalog.org/publication/PGP000237/). PRS scores were calculated using the PLINK (v1.9) software [53] based on the posterior effect sizes of the summary statistics generated by the PRS-CS software and reported effect sizes of the PGS catalog SNPs.

### Statistical analyses

All analyses were performed using R version 4.0.0 [54]. Descriptive statistics were used to summarise and compare data across the cohorts. To achieve normality of the lipid variables, inverse normal transformations were performed using the *rntransform* function in the R package version 1.8–0 GenABEL (https://CRAN.R-project.org/package=GenABEL). Regressions (logistic for binary dependent variables, linear for continuous), controlling for age, sex and 10 genetic principal components (PCs) were performed to assess if the ELPRS (+ / − *APOE* locus) was associated with (i) the presence/absence of MetS and/or (ii) the individual components of MetS. Sex-stratified analyses were also performed in the UKBB but not in other cohorts due to smaller sample sizes.

Fixed and random effect models based on the inverse variance method were used for meta-analysis as implemented in the R package metafor [55]. The significance of the pooled odds ratio (OR) was determined by the *Z*-test. The *I*^2^ statistic was used to estimate the percentage of variation across the results due to study heterogeneity, rather than sampling error. No significant heterogeneity was defined as an *I*^2^ value of less than 50% and/or a *p*-value < 0.05.

## Results

### Sample characteristics

**Fig. 2:**
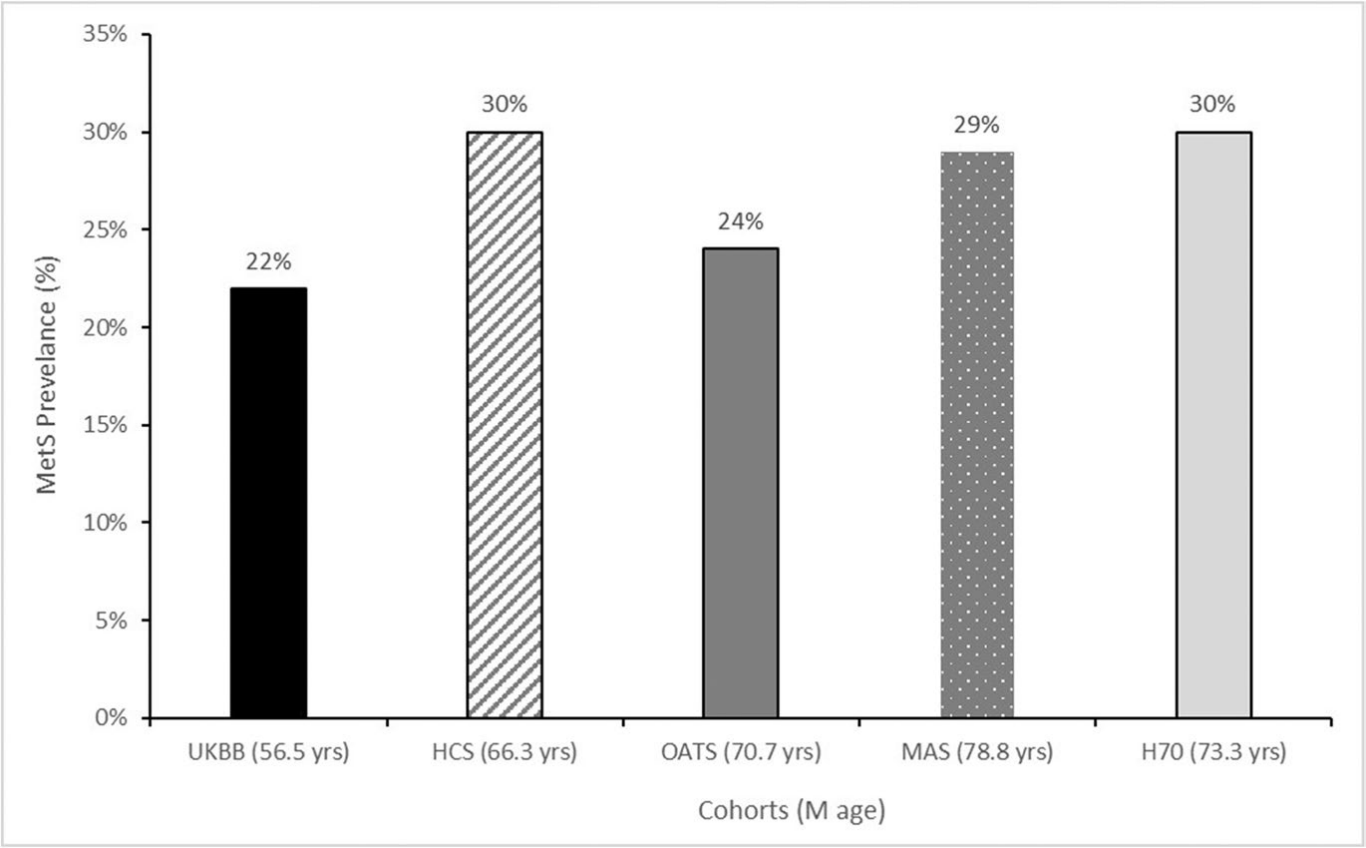
MetS prevalence across the UKBB and the replication cohorts: HCS, OATS, Sydney, MAS and H70 cohorts

The sample characteristics of the five studies are described in Table 1. The prevalence of MetS was 22% in the UKBB cohort. The replication cohorts had a prevalence of 30%, 24%, 29% and 30% in the HCS, OATS, MAS and H70 study respectively (Fig. 2).

**Table 1:** Descriptive characteristics of participants across the UKBB and the replication cohorts: HCS, OATS, Sydney MAS and H70 cohorts

Cohort	UKBB	HCS	OATS	MAS	H70
Sample size (*N*)	407 807	2122	539	925	2273
Age range (years)	40–69 (56.5 ± 7.9)	55–85 (66.3 ± 7.5)	65–90 (70.7 ± 5.4)	70–90 (78.8 ± 4.8)	70–93 (73.3 ± 4.7)
*n*, (%) males	187 365 (46)	1042 (49)	217 (40)	417 (45)	417 (45)
*APOE4* carrier, *n* (%)	99 138 (24)	604 (28)	145 (23)	213 (23)	680 (30)
Systolic (mmHg)	140 ± 19.65	135 ± 8.44	141 ± 21.18	146 ± 20.17	147 ± 21.79
Diastolic (mmHg)	82 ± 10.66	79 ± 9.88	83 ± 14.23	83 ± 10.61	81 ± 11.02
Antihypertensive use, *n* (%)	46,585 (25)	48 (3)	285 (48)	597 (65)	976 (43)
Serum glucose (mmol/L)	5.12 ± 1.21	5.19 ± 1.20	4.77 ± 1.11	5.88 ± 1.22	5.91 ± 1.55
Antidiabetic treatment, *n* (%)	2561 (1)	124 (7)	39 (6)	90 (10)	220 (10)
Serum triglycerides (mmol/L)	1.76 ± 1.02	1.39 ± 0.99	1.28 ± 0.68	1.44 ± 0.44	1.66 ± 0.50
HDL-cholesterol (mmol/L)	1.45 ± 0.38	1.34 ± 0.37	1.41 ± 0.42	1.44 ± 0.44	1.66 ± 0.50
Waist (cm)	90.34 ± 13.48	96.39 ± 13.35	94.03 ± 12.72	96.58 ± 13.01	92.79 ± 12.70
Presence of MetS/*n* available, (%)	73,167/334157 (22)	543/1809 (30)	150/539 (24)	271/925 (29)	687/2273 (30)

*UKBB*, UK Biobank; *HCS*, Hunter Community Study; *OATS*, Older Australian Twins Study; *MAS*, Sydney Memory and Ageing Study; *H70*, Gothenburg H70 birth cohort; *HDL*, high-density lipoprotein; *MetS*, metabolic syndrome. Raw mean values ± standard deviation are reported for continuous variables and frequencies (%) for categorical

### ELPRS and MetS

Logistic regressions were performed to ascertain whether ELPRS was associated with MetS. For the 90^th^ percentile survival case/control analysis, as expected, the ELPRS was inversely and highly significantly associated with MetS in the UKBB (OR = 0.94, *p* = 1.84 × 10^–42^) (Table 2). Sex and age were also significantly associated with MetS in the UKBB (*p* = 2.68 × 10^–9^ & *p* = 9.31 × 10^–82^ and respectively) (Table S1). Highly significant results were observed for all the components of MetS, with all results in the expected direction (Table 2). Secondary analyses using alternative PRSs (99^th^ percentile; 330 variants) gave comparable results in the same direction (Tables S1–S4); hence, only the results for the 90^th^ percentile for survival are presented from herein.

**Table 2:** Associations of ELPRS (based on cases who had lived to an age above the 90^th^ percentile for survival and controls who had died at or before the 60^th^ percentile) with MetS and its components in the UK Biobank (UKBB) and the meta-analysis results for the four replication cohorts

		UKBB association results				Replication meta-analysis results				
Metabolic measures	*n*^a^	*β* ± SE	*OR*	*p*val	*n*^b^	*β* ± S.E	*OR*	*p*val	*I*^2^	Heterogeneity *p*val
Systolic blood pressure (mmHg)	380,953	* − *0.023 ± 0.002	0.977	**1.48 × 10**^**–54**^	5781	* − *0.005 ± 0.013	0.995	0.691	0	0.409
Diastolic blood pressure (mmHg)	380,961	* − *0.022 ± 0.002	0.978	**6.70 × 10**^**–42**^	5782	* − *0.001 ± 0.013	0.999	0.979	31.34	0.224
Antihypertensive use	187,176	* − *0.109 ± 0.005	0.897	**3.51 × 10**^**–83**^	5589	* − *0.116 ± 0.036	0.890	**0.001**	22.73	0.275
Serum glucose (mmol/L)	355,611	* − *0.015 ± 0.002	0.985	**5.84 × 10**^**–18**^	5498	* − *0.001 ± 0.013	0.999	0.989	0	0.965
Antidiabetic use	187,176	* − *0.114 ± 0.020	0.892	**1.76 × 10**^**–8**^	5124	* − *0.125 ± 0.052	0.882	**0.016**	0	0.674
Serum triglycerides (mmol/L)	388,133	* − *0.016 ± 0.002	0.984	**1.37 × 10**^**–24**^	5764	* − *0.006 ± 0.012	0.994	0.595	0	0.609
HDL-cholesterol (mmol/L)	355,864	0.024 ± 0.002	1.024	**3.35 × 10**^**–55**^	5762	0.025 ± 0.011	1.025	**0.029**	0	0.395
Waist Circumference (cm)	407,126	* − *0.026 ± 0.001	0.974	**9.07 × 10**^**–77**^	5827	* − *0.012 ± 0.012	0.988	0.324	31.27	0.225
MetS data available	334,157	* − *0.058 ± 0.004	0.944	**1.84 × 10**^**–42**^	5481	* − *0.069 ± 0.031	0.933	**0.028**	53.14	0.094

^a^Sample available for a particular analysis. ^b^Sample totals of 4 replication cohorts. *UKBB*, UK Biobank; replication cohorts are *HCS*, Hunter Community Study; *OATS*, Older Australian Twins Study; *MAS*, Sydney Memory and Ageing Study; *H70*, Gothenburg H70 birth cohort; *HDL*, high-density lipoprotein; *MetS*, metabolic syndrome. Linear regressions were used for continuous variables and logistic regressions for binary. Beta coefficients and their standard errors are reported. *p*-values ≤ 0.05 are bolded. The *I*^2^ statistic was used to estimate the percentage of variation across the results due to study heterogeneity, rather than sampling error. No significant heterogeneity was defined as an *I*^2^ value of less than 50% and a *p*-value > 0.05

**Fig. 3:**
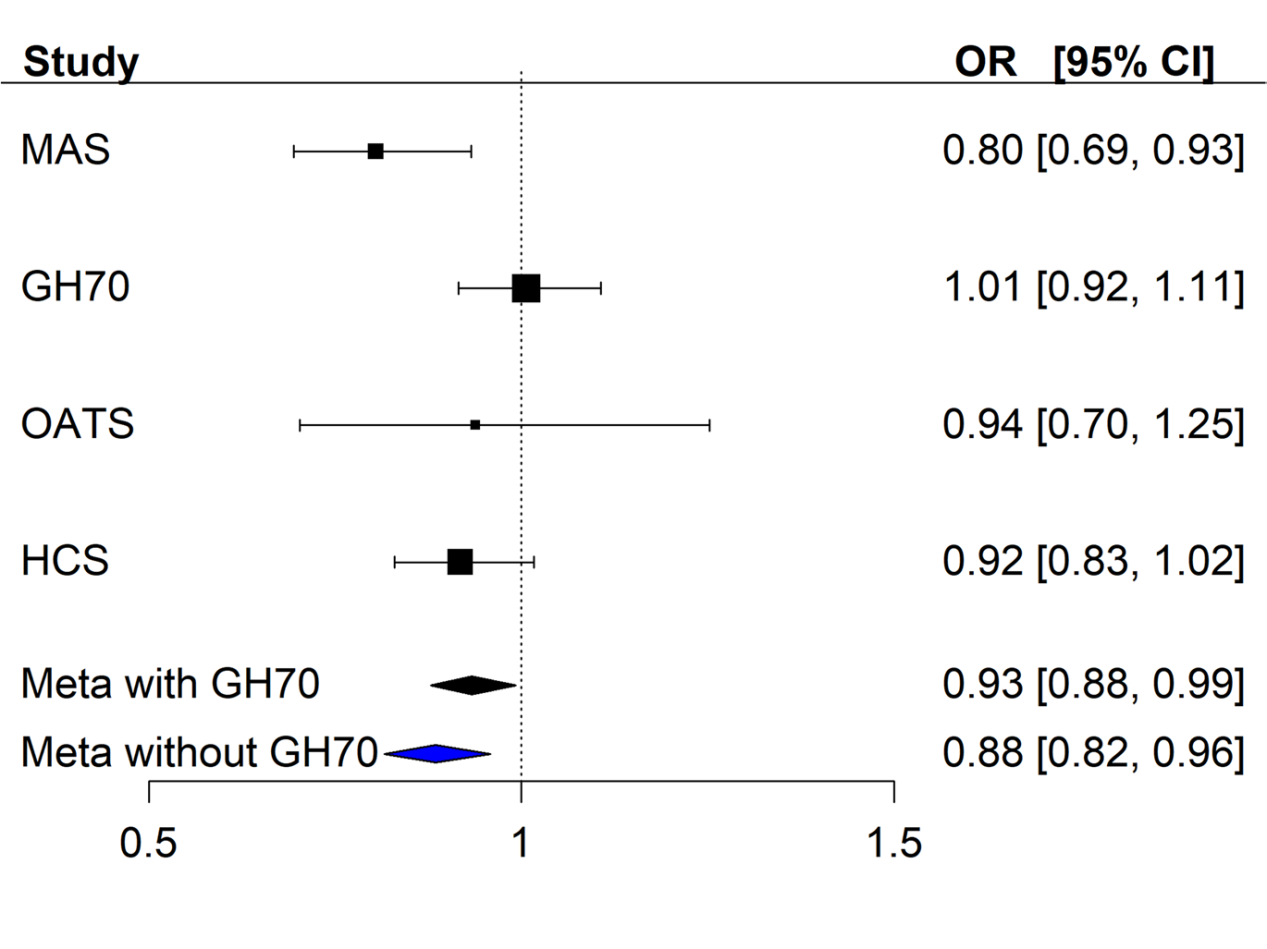
A forest plot illustrating the association of the replication cohorts with the polygenic risk score for EL and MetS

A meta-analysis of the four replication cohorts showed an inverse association of MetS with the ELPRS (OR = 0.93, 95% CI = 0.88–0.99, *p* = 0.028) (Table 2, Fig. 3). Heterogeneity between the smaller replication studies was not significant. The OR for the Swedish H70 cohort was positive, approximately one, which was opposite to the other studies (OR < 1). When only the Australian cohorts were meta-analysed, the results were strengthened (OR = 0.88, 95% CI = 0.82–0.96, *p* = 0.003) (Fig. 3 & Table S6). Meta-analysis of the individual components of MetS (Table 2) using the four replication cohorts showed no or low heterogeneity. Three of the eight components showed significant or nominal associations with ELPRS: the use of antihypertensives (OR = 0.89, *p* = 0.001) and antidiabetics (OR = 0.88, *p* = 0.016) as well as HDL-cholesterol (OR = 1.03, *p* = 0.029). The pooled effect sizes for MetS and its components from the replication meta-analysis were comparable and in the same direction to the UKBB (e.g. MetS *β* =  − 0.069 vs *β* =  − 0.058 respectively) (Table 2).

Individual cohort results examining the associations between ELPRS and MetS are available in the Supplementary (Tables S8 & S9). Sydney MAS was nominally significant (OR = 0.81, *p* = 0.038) whilst the other replication cohorts did not reach statistical significance but were generally in similar directions to the UKBB results. Within these smaller studies, no components of MetS were significantly associated with the ELPRS.

Additional analyses excluding the *APOE* locus in the UKBB (Table S1), in the replication meta-analysis (Table S5 & S7) and in the individual cohorts (Table S9) did not markedly change the results.

### ELPRS and MetS sex-stratified analyses

Due to the large sample size of the UKBB, interaction analysis between sex and ELPRS and the sex-stratified analyses were only performed in this cohort. The sex by ELPRS interaction was not significant (− 0.021 ± 0.08, *p* = 0.150). However, given the known sex differences for MetS and longevity [56], sex-stratified analyses were undertaken using logistic regressions and adjusted for age (Table 3), with both sexes showing significant inverse relationships with MetS and the ELPRS. Removal of the *APOE* locus did not appreciably alter the results (Table S10). Age was statistically significant in both sexes.

**Table 3:** Sex-stratified associations of ELPRS with MetS in the UKBB

	*n*	*β* ± *SE*	*OR*	*p*
*Females only*	170,456	− 0.052 ± 0.005	0.949	**9.04 × 10**^**–19**^
*Males only*	163,701	− 0.063 ± 0.006	0.939	**2.67 × 10**^**–26**^

*ELPRS*, exceptional longevity polygenic risk score; *UKBB*, UK Biobank; *MetS*, metabolic syndrome. Linear regressions, adjusted for age, with independent variable = ELPRS and dependent variable = MetS

*P*-values ≤ 0.05 are bolded

## Discussion

This study supports the hypothesis that individuals with a high polygenic risk score (PRS) for exceptional longevity (EL) have a greater likelihood of a healthy metabolic profile of MetS and its sub-components.

MetS prevalence ranged from 22 to 30% across the five cohorts. The UKBB’s prevalence is consistent with previous work by Lind on the same cohort, who found a MetS occurrence of 20.5% [44]. We used the same MetS definition as Lind and the observed small difference in incidence may reflect the larger number of UKBB samples used in our study (*n* = 291,107 [Lind] versus *n* = 334,157 [current study]). However, in Sydney MAS, MetS prevalence was considerably lower than that reported previously (current study 29% versus 54% [57]). This difference is likely explained by the criteria used to calculate MetS. This investigation utilized the harmonized National Cholesterol Education Program (NCEP) definition [44]; whereas, Samaras et al. [57], applied the International Diabetes Federation (IDF) criteria that has central obesity (defined by waist circumference according to ethnicity) as a pre-requisite and uses lower waist measurement cut-offs. MetS prevalence varies greatly amongst different ethnicities, ranging from 11 to 41% in India [58], ~ 34% in the USA and ~ 32% in Koreans [59]. Despite differences in methodology, such as MetS criteria, MetS prevalence is increasing worldwide with age [60]. This was also observed in this study; however, one replication cohort, OATS (mean age 71 years), had almost as low prevalence (24%) as the youngest (mean age 57 years) cohort, the UKBB (22%).

High polygenic risk for EL was significantly associated with MetS in the UKBB. However, a meta-analysis of the replication cohorts showed only a nominally significant association (*p* = 0.028), which was strengthened when the Swedish H70 Birth cohort was removed (*p* = 0.003). The magnitude of the effect sizes of the primary and replication analyses was similar confirming the association of polygenic risk for EL with MetS. The nominal significance observed in the smaller cohorts is most likely due to the much smaller sample size and, thus, the larger standard error.

In addition, all of the components of MetS were highly significant in the UKBB, whereas none of the components reached significance in the individual replication cohorts. However, the results of a meta-analysis including these four replication cohorts indicated the following MetS components as statistically or nominally (*p* < 0.05) significant: the use of antihypertensives, antidiabetics (inverse association) and HDL-cholesterol (positive association). The relationships between medication and the EL PRS are in the hypothesized direction, as individuals that have a high ELPRS would be expected less likely to have diabetes and hypertension and hence would not be reliant on such medications. The association between high levels of HDL-cholesterol and high genetic risk for EL is also not unexpected, given the known relationship between elevated HDL levels and good health. Overall, these results suggest that not only MetS but also some of the individual components are also associated with the ELPRS.

Interestingly, previous work on exceptionally long-lived individuals in the Sydney Centenarian Study showed a higher HDL-cholesterol PRS was nominally associated with longevity [61]. Recently, another study from the USA observed a positive correlation with ageing and higher HDL-cholesterol levels in the exceptionally long-lived (≥ 90 years, *n* = 291) [62]. Furthermore, genetic correlation analyses by Deelen et al., indicated that two of the components of MetS, HDL-cholesterol and waist circumference, were significantly correlated with EL [38], providing evidence that there are common genetic variants underlying these measures and longevity. A recent analysis found that extremely long-lived individuals have a similar burden of genetic disease risk as the general population except for significantly lower polygenic risk scores for coronary artery disease (CAD) and Alzheimer’s disease (AD) and higher scores for cognitive function and parental longevity [63]. However, this study did not examine MetS. Our study extends this prior work by finding a lower prevalence of MetS in individuals with a high polygenic risk for EL.

The use of two alternate EL PRS, one based on a more extreme phenotype (cases that had lived to an age ≥ 99^th^ percentile of survival, [38]) and the other using 330 variants [52], gave similar results. This is despite the latter PRS being based on an analysis using a cohort of centenarians that had remained cognitively healthy [52] whereas our cohorts comprise a mixture of individuals, both healthy and unhealthy.

No interaction between sex and MetS was observed in the UKBB. This question was not examined in the other cohorts due to their small sample sizes. In contrast to our null findings in the UKBB there is evidence supporting sex specific differences in the development of MetS, largely arising from hormonal regulation of body fat distribution and dysglycemia [56, 64]. Another study found that elevated BMI, low HDL-cholesterol, increased waist circumference and hyperglycemia were significantly larger contributors to the metabolic syndrome in women as opposed to hypertension and elevated triglycerides in men [65]. Furthermore, as the prevalence of each MetS criterion differs with age and sex, it has been reported that MetS development may be affected by the presence of different combinations of MetS criteria [66]. Therefore, further examination of sex and MetS criteria, such as at different stages of the lifespan, may be warranted.

The major strength of this study is the harmonized definition of MetS utilised across the five independent cohorts ranging in age from 40 to 93 years. The UKBB was large in sample size providing high statistical power. Limitations include the lack of fasting blood metabolic measurements in the primary cohort (UKBB), whereas fasting bloods were attained in the replication cohorts. However, HDL-cholesterol is not majorly affected by fasting. Additionally, in the UKBB analyses, glucose and triglyceride measurements were adjusted for the amount of time between blood collection and their last meal. Participants with data missing for three or more of the five MetS criteria were not included in the current study; hence, our estimate of the prevalence of MetS may be imprecise. Another constraint is the smaller size of the replication cohorts, which decreases the available statistical power. Moreover, the replication cohorts contain individuals of an older age compared to the UKBB, which introduces the issue of survivor bias into our analyses. MetS is more likely to affect life expectancy, and hence, the participants of the replication studies may represent survivors, potentially confounding the analyses. Ideally, replication and validation of our primary analysis should be completed in cohorts of a similar age. Interestingly, MetS prevalence in a centenarian cohort was lower when compared to the replication cohorts utilised in this study (data not shown). This suggests that most individuals with MetS do not survive to a greater age and for any that do it may be due to protective genetic factors, environmental influences or a combination of both as well as epigenetic changes that provide a survival advantage. The samples used in this analysis were of European ancestry, and hence, these results may not generalize to other ethnic/racial populations [15]. Finally, the PRS provides an overall estimate of the genetic predisposition for EL. In general, a limitation of the use of PRSs is that they currently explain only a small proportion of the observed variance, which is also true for the current analysis. Furthermore, there are different methods of calculating a PRS [67], which may lead to inconsistencies in results observed across studies. Thus, a standardised method would be best. An open resource of published polygenic scores (https://www.PGSCatalog.org) has been developed and utilising this in future could end the debate regarding the optimum PRS method to use [68]. Finally, the choice of summary statistics for use in calculating the PRS may also differ from study to study. This study attempted to appraise a few different PRS by calculating two PRS based on different case age cut-offs from one GWAS and computing a PRS based on another study. Future EL genetic studies will continue to identify new variants and improve the variance explained by the ELPRS.

This study has shown the importance of maintaining a strong and healthy metabolic profile in terms of its benefits for one’s longevity. The recommendations for achieving this are by incorporating as many positive lifestyle changes as possible. For example, eating a healthy diet with plenty of natural wholegrain foods and limiting sugars and saturated fats. Other examples include not smoking and avoiding excessive amounts of alcohol. The benefits of increased physical activity and managing a healthy body weight are paramount to a strong metabolic profile that promotes longevity.

## Conclusions

In summary, these findings suggest individuals who have high polygenic risk for EL have a lower prevalence of MetS and its components. Our results suggest that genetic variants that contribute to lifespan may also be involved in maintaining a healthy metabolic profile. Thus, further research into unravelling the function of longevity-related variants may assist in a better understanding of the underlying biological mechanisms contributing to MetS and health span. Ultimately, such data could lead to new strategies and treatments to foster metabolic health in older adults. Whether these results also apply to individuals of non-European ancestry should be addressed in future studies. Further work will explore the prevalence of MetS in centenarians and supercentenarians, which may further reveal the extent of the importance of MetS on ageing successfully.

## Supplementary Information

Below is the link to the electronic supplementary material.

Supplementary file1 (DOCX 47.3 KB)
